# Parasitic Pneumonia and Lung Involvement

**DOI:** 10.1155/2014/874021

**Published:** 2014-06-09

**Authors:** Attapon Cheepsattayakorn, Ruangrong Cheepsattayakorn

**Affiliations:** ^1^10th Zonal Tuberculosis and Chest Disease Center, Chiang Mai, Thailand; ^2^10th Office of Disease Prevention and Control, Department of Disease Control, Ministry of Public Health, Chiang Mai 50100, Thailand; ^3^Department of Pathology, Faculty of Medicine, Chiang Mai University, Chiang Mai 50200, Thailand

## Abstract

Parasitic infestations demonstrated a decline in the past decade as a result of better hygiene practices and improved socioeconomic conditions. Nevertheless, global immigration, increased numbers of the immunocompromised people, international traveling, global warming, and rapid urbanization of the cities have increased the susceptibility of the world population to parasitic diseases. A number of new human parasites, such as *Plasmodium knowlesi*, in addition to many potential parasites, have urged the interest of scientific community. A broad spectrum of protozoal parasites frequently affects the respiratory system, particularly the lungs. The diagnosis of parasitic diseases of airway is challenging due to their wide varieties of clinical and roentgenographic presentations. So detailed interrogations of travel history to endemic areas are critical for clinicians or pulmonologists to manage this entity. The migrating adult worms can cause mechanical airway obstruction, while the larvae can cause airway inflammation. This paper provides a comprehensive review of both protozoal and helminthic infestations that affect the airway system, particularly the lungs, including clinical and roentgenographic presentations, diagnostic tests, and therapeutic approaches.

## 1. Introduction

Protozoal and helminthic parasitic pneumonias and lung involvement are common in the tropics [1] with few exceptions; they most commonly occur in the western world and are diseases of immunocompromised hosts [2]. In USA,* Toxoplasma gondii* pneumonia is observed most frequently in acquired-immunodeficiency-syndrome (AIDS) patients. Pulmonary strongyloidiasis is identified in patients with chemotherapy or glucocorticoids treatment and is endemic in the Southeastern USA, whereas Ascaris and hookworm infestations may be present with eosinophilia and pulmonary infiltrates during the larval migration stage [2]. Nevertheless, protozoal infestations usually do not cause blood and tissue eosinophilia except helminthic infestations [1]. In 1932, Loffler described the first four cases with minimal respiratory symptoms, peripheral blood eosinophilia, and pulmonary infiltrates in the chest roentgenographs [3]. The eosinophilic lung diseases that are particularly prevalent in the tropics are frequently related to parasitic infestations [4], whereas* Entamoeba histolytica*,* Paragonimus*, and* Dirofilaria* lung involvement are less common [2].

## 2. Pulmonary Malaria

The four types of malarial parasites are* Plasmodium falciparum*,* Plasmodium malariae*,* Plasmodium vivax*, and* Plasmodium ovale*, and the protozoa of the genus* Plasmodium* cause Malaria and are primarily transmitted by the bite of an infected female* Anopheles* mosquito to infect humans [5]. The malaria parasites then infect human hepatocytes in the forms of sporozoites, schizonts, and merozoites, respectively [6]. This stage is called “human liver stage” which takes approximately 4.5 days for* Plasmodium falciparum* [6]. In case of infection with* Plasmodium vivax*, one of the two forms (*Plasmodium vivax* and* Plasmodium ovale*) of relapsing malaria to infect humans, and is the most prevalent in Southeast Asia and South America, has ability to become dormant in the liver (“hypnozoite”) and is able to be reactivated after months or years contributing to an attack of intraerythrocytic stage malaria despite the absence of mosquito bites [6, 7]. The merozoites then burst out from the hepatocytes and infect human erythrocytes in the form of ring form, trophozoites, schizonts, and merozoites again [6]. This cycle takes approximately 43–48 hours for the* Plasmodium falciparum*, and the cycle is able to develop clinical symptoms [6]. In another cycle, the intraerythrocytic ring form transforms to intraerythrocytic gametocyte before infecting the mosquito [6]. This stage takes approximately 9 days for* Plasmodium falciparum* [6]. Human liver stage and human intraerythrocytic stage are asexual stages [6]. After mosquito ingestion of the human malaria-infected blood (intraerythrocytic gametocytes), the gametocyte transforms to microgametocyte and microgametocyte which take approximately 15 minutes and then transform to diploid zygote in one hour for* Plasmodium falciparum*, respectively, in the mosquito's midgut [6]. The diploid zygote then transforms to ookinete in approximately 12–36 hours for* Plasmodium falciparum* before transforming to oocysts and sporozoites in the mosquito's salivary gland before transmission of the sporozoite to humans by mosquito biting [6]. The malaria parasite stage in the mosquito is called “mosquito stage or sexual stage” [6]. In most cases, the incubation period varies from 7 to 30 days [7]. The shorter incubation periods are demonstrated in* Plasmodium falciparum*, while the longer ones are observed in* Plasmodium malariae* [7]. Returned travelers should remind their healthcare providers of any travel in areas where malaria occurs during the past 12 months [7]. The main finding of patients with falciparum malaria which is the most deadly type of malaria infection is sequestration of erythrocytes containing mature forms of* Plasmodium falciparum* in the microvasculature of the organs and is quantified by measurement of* Plasmodium falciparum* specific histidine-rich protein 2 (PfHRP2) using a quantitative antigen-capture enzyme-linked immunosorbent assay [8]. Gas exchange is significantly impaired in patients with severe malaria [9].

In patients with uncomplicated malaria, the classical (but infrequently observed) malaria attack lasts 6–10 hours that consists of a cold stage (sensation of cold, shivering), a hot stage (fever, headaches, vomiting, and seizures in young children), and a sweating stage (sweats, return to normal temperature, tiredness) [7]. Classically, the attacks occur on every second day with the “tertian” malaria parasites (*Plasmodium falciparum*,* Plasmodium vivax*, and* Plasmodium ovale*) and on every third day with “quartan” parasite (*Plasmodium malariae*) [7]. More commonly, the patients present with a combination of the following symptoms: general malaise, fever, chills, headaches, body aches, nausea, and vomiting [7]. The physical findings may include elevation of body temperatures, weakness, perspiration, increased respiratory rate, mild jaundice, enlargement of the liver, and enlargement of the spleen [7]. In patients with severe malaria, the clinical manifestations include cerebral malaria (abnormal behavior, impairment of consciousness, seizures, coma, or other neurological abnormalities), severe anemia due to hemolysis, hemoglobinuria due to hemolysis, abnormal blood coagulation, low blood pressure, acute renal failure, hyperparasitemia (more than of the malaria parasite-infected erythrocytes, hypoglycemia, metabolic acidosis, and acute respiratory distress syndrome) [7]. Recurrent infections with* Plasmodium falciparum* may result in severe anemia [7]. Nephrotic syndrome can result from repeated infections with* Plasmodium malariae* [7]. Hyperreactive malarial splenomegaly (or called “tropical splenomegaly syndrome”) is marked by the much enlarged spleen and liver, anemia, abnormal immunological findings, and a susceptibility to other infections and is attributed to an abnormal immune response to repeated malarial infections [7].* Plasmodium falciparum* may cause more severe disease in mother and may lead to premature delivery or delivery of a low-birth-weight baby [7]. Neurological defects may occasionally persist following cerebral malaria, particularly in children [7]. On rare occasions,* Plasmodium vivax* can cause rupture of the spleen [7]. The most common presentations of falciparum malaria in the study were fever, followed by jaundice, renal involvement, cerebral malaria, severe anemia, bleeding manifestation, and hypoglycemia [10]. The gold standards for the diagnosis of malaria are light microscopic examination of thin and thick stained blood smears [1, 11]. Additional laboratory findings may include mild anemia, mild thrombocytopenia, elevation of aminotransferase, and elevation of serum bilirubin [7]. Human urine and saliva PCR detection of* Plasmodium falciparum* have been introduced [11]. In severe falciparum malaria, the roentgenographic presentations include diffuse interstitial edema, pulmonary edema, pleural effusion, and lobar consolidation [11]. Sanklecha et al. reported three cases of childhood falciparum malaria in a family and revealed that two cases demonstrated bilaterally fluffy pulmonary infiltrates, whereas the remaining case showed normal chest roentgenogram [12]. Chest roentgenograms in three cases of malaria with sickle cell anemia also reported that all reported patients demonstrated bilaterally pulmonary infiltrates [13]. Chest roentgenograms are usually nonspecific, but they should be recognized in high endemic areas of malaria [13]. Chest roentgenogram in a patient with* Plasmodium vivax* malaria demonstrated diffuse bilateral alveolar opacities which indicated acute respiratory distress syndrome [14]. Three cases of ARDS with* Plasmodium vivax* malaria were also reported in India and one case was demonstrated with bilateral perihilar infiltrates, one case with bilateral diffuse extensive opacities, and another case with bilateral basal ground glass opacities on chest roentgenograms [15]. Pulmonary edema is universal finding at autopsy [16]. The alveoli are filled parasite-red blood cells, nonparasitic red blood cells, neutrophils, and pigment-laden macrophages [16]. In many severe cases, alveoli are lined with a laminated periodic acid-schiff (PAS) positive membrane which finally destroys and incorporated the alveolar wall within it [16]. This is associated with abundant edematous fluid and pulmonary vasodilatation and may have a marked inflammatory infiltrate [16]. There is hyaline membrane formation in the alveoli that indicates leakage of proteinaceous fluid, particularly in falciparum malaria [16]. The majority of blood vessels showed parasite-red blood cell sequestration in the septal capillaries and small blood vessels in the lung of the severe cases [17]. Mononuclear cell-pigment laden macrophages were seen admix with parasite-red blood cells in the microvessels of alveolar septa [17]. Pulmonary vascular occlusion could occur in both patients with uncomplicated malaria and those with severe malaria [9]. A recent study demonstrated that platelet-activating-factor receptor activation was critical in the pathogenesis of pulmonary damage associated with* Plasmodium berghei* ANKA strain infection in a mice model [18]. About 60% of these infected mice had hypoxemia, dyspnea, pleural effusion, airway obstruction, pulmonary edema, and pulmonary hemorrhage [18]. There is little knowledge about the pathogenesis of malaria-associated acute lung injury and adult respiratory distress syndrome (ARDS) [19]. Increased endothelial permeability and inflammatory mediators may play an important role, whereas parasite sequestration may take a minor role that supported by elevation of level of vascular endothelial growth factor found in mice model [19]. In* in vitro* studies,* Plasmodium falciparum* merozoite proteins could increase pulmonary endothelial permeability, whereas* Plasmodium falciparum* infected-red blood cells did not demonstrate the same properties indicating that the effects of the malaria parasites on the pulmonary endothelium probably mediated the activity of Src-family kinases [19]. Increased water content in infected mice lungs was identified and contributed to the development of pulmonary edema [19]. A study in DBA2 mice infected with* Plasmodium berghei* K173 demonstrated that proteins and inflammatory cells mainly CD4+ and CD8+ lymphocytes, monocytes, and neutrophils accumulated in the lungs of infected mice [19]. Van den Steen et al. measured levels of cytokines and chemokines associated with ARDS and demonstrated an expression of tumor-necrosis-factor-*α*, interferon-*γ*, CXCL10 and CXCL11, as well as neutrophil and monocyte chemo-attractant chemokines (CCL2, KC) in the lungs [19]. Pulmonary manifestations of uncomplicated malaria may include subclinical impairment of lung function, such as impaired alveolar ventilation, reduced gas exchange, and increased pulmonary phagocytic activity [19]. Despite the fact that ARDS is most commonly identified as a complication in patients with* Plasmodium falciparum* malaria, ARDS patients with* Plasmodium vivax*,* Plasmodium ovale*, and* Plasmodium knowlesi* were also reported [19]. The greatest severity and frequency of ARDS in malaria cases are due to* Plasmodium falciparum* and could be partially attributed the resetting and sequestration of parasite-infected red blood cells in the pulmonary microcirculation [19]. Heavy parasitemia and white blood cell agglutinates are associated with ARDS in patients with* Plasmodium vivax* malaria and principally could be due to dysregulation of cytokine production [19]. Increased parasitemia in patients infected with* Plasmodium knowlesi* indicates that parasite-specific effects increase pulmonary capillary permeability but could contribute to hypoxemia and metabolic acidosis [19]. Intravenous chloroquine is the drug of choice for chloroquine-susceptible* Plasmodium falciparum* infections and those rare cases of life-threatening malaria caused by* Plasmodium vivax*,* Plasmodium malariae*, and* Plasmodium ovale* [1, 11]. A point mutation in the* Plasmodium falciparum* chloroquine-resistance transporter (PfCRT) gene is responsible for chloroquine-resistant falciparum malaria [20], whereas disappearance of the K76T mutation in PfCRT is associated with chloroquine susceptibility [21]. Oral artemisinin-based combination therapies (artesunate + mefloquine, artesunate + sulfadoxine-pyrimethamine artesunate + amodiaquine, or artemether + lumefantrine) are the best antimalarial drugs [22, 23]. Additionally, the World Health Organization (WHO) recommends oral treatment of dihydroartemisinin plus piperaquine as soon as the patients are able to take oral medication but not before a minimum of 24 hours of parenteral treatment [24]. The WHO recommended that intravenous artesunate can be used preferentially over quinine for the treatment of severe malaria caused by any* Plasmodium* species in both children and adults [25]. Oral artemisinin-based combination therapies have also demonstrated equivalent (if not better) efficacy in the treatment of uncomplicated malaria caused by all* Plasmodium* species and chloroquine-resistant* Plasmodium vivax* in both children and adults [25]. Hence, conventional therapeutic regimens continue to be efficacious [25]. Treatment of relapsing malaria should follow treatment of the first attack [7]. Insecticide-treated bed nets in which insecticide is incorporated into the net fibers are evidenced to be the best way to prevent malaria [26]. It is demonstrated that RTS, S/ASO2, a vaccine, has demonstrated promising results in endemic areas [26]. Dexamethasone may be included in adjunct chemotherapy with anti-inflammatory drugs due to inhibition of infiltration of CD8+ T-cells and macrophages into the lungs in rodent model malaria associated ARDS [19].

## 3. Pulmonary Amoebiasis

*Entamoeba histolytica*, a well-recognized pathogenic amoeba, is associated with both intestinal and extraintestinal infections [27]. Both cysts and trophozoites are passed in human feces [27]. Cysts are typically identified in formed stool, while trophozoites are typically found in diarrheal stool [27]. Humans are infected by ingestion of mature cysts in fecally contaminated food, water, or hands [27]. Excystation occurs in the small bowel and trophozoites are released, which migrate to the large bowel [27]. The trophozoites multiply by binary fission and produce cysts, and both stages are passed in the feces [27]. Due to the protection conferred by their walls, the cysts are able to survive days to weeks in the external environment and have potential for transmission [27]. In contrast, trophozoites passed in the diarrheal stool are rapidly destroyed once outside the body, and if ingested they would not survive when they are exposed to the gastric environment [27]. Surprisingly, in many patients, the trophozoites remain confined to the intestinal lumen (noninvasive infection) of the individuals who are asymptomatic carriers and pass cysts in their stool [27]. In some cases, the trophozoites invade the intestinal mucosa (intestinal disease), or the bloodstream, extraintestinal sites such as the liver, lungs, and brain, resulting in hepatic amoebiasis, pulmonary amoebiasis, cerebral amoebiasis, and so forth [27]. Pulmonary amoebiasis caused by the protozoan parasite,* Entamoeba histolytica*, occurs mainly by the extension from the amoebic liver abscess [28, 29]. The invasive and noninvasive forms,* Entamoeba histolytica* and* Entamoeba dispar*, respectively, have been established [27].* Entamoeba histolytica* is identified with ingested red blood cells (erythrophagocytosis) [27]. Transmission of cysts or trophozoites can occur through exposure to fecal matter during sexual contact [27]. The immune mechanism that explains why only a subset of* Entamoeba histolytica*-exposed persons develops clinical disease is not fully understood [30]. The effect of microbiota on immune response to* Entamoeba histolytica* and its virulence is not yet known [30]. The presence of cysts or trophozoites of amoeba in the stool does not imply that the disease is caused by* Entamoeba histolytica* as other two non-pathogenic species found in humans (*Entamoeba dispar* and* Entamoeba moshkovskii*) are morphologically indistinguishable but can be rapidly, accurately, and effectively diagnosed by a single-round PCR [1, 31, 32]. Other diagnostic methods include culture of* Entamoeba histolytica*, enzyme-linked immunosorbent assay (ELISA), indirect fluorescent antibody test (IFAT), and indirect hemagglutination test (IHA) [1, 31, 32]. A combination of serological tests with identification of the parasite by PCR or antigen detection is the best diagnostic approach [33]. Active trophozoites of* Entamoeba histolytica* can be identified in sputum or pleural specimen [1]. Physical examination reveals fever, chest pain, tender hepatomegaly, and cough are indicated pleuropulmonary amoebiasis [1]. Some patients may present with hemoptysis, expectoration of anchovy sauce-liked pus, respiratory distress, and shock [1]. Chest roentgenographic findings include pleural effusion, basal pulmonary involvement, and elevation of hemidiaphragm [1]. Metronidazole is the treatment of choice [34]. Diloxanide furoate, a luminal amoebicidal drug, can eliminate intestinal* Entamoeba* cysts [35]. Lactoferrin and lactoferricins, amoebicidal drugs, can be coadministered with a low dose of metronidazole to reduce metronidazole toxicity [35]. Identification of possible vaccine candidates against amoebiasis is in progressive studies [36].

## 4. Pulmonary Leishmaniasis

Pulmonaryor visceral leishmaniasis, also called “Kala azar” is caused by* Leishmania donovani* and* Leishmania chagasi* or* infantum* [37], is transmitted by various species of* Phlebotomus*, a type of sand fly [38].* Leishmania amastigotes* can be identified in pulmonary septa, alveoli, and the BAL fluid [39, 40]. Pleural effusion, mediastinal lymphadenopathy, and pneumonitis have been reported in HIV-infected patients with visceral leishmaniasis and lung transplant patients [39, 40]. The expansion of the HIV-infection/AIDS epidemic over leishmaniasis, particularly visceral leishmaniasis endemic regions, has increased the number of coinfected patients [41] indicating that visceral leishmaniasis is an opportunistic disease in HIV-infected/AIDS patients although not yet considered an AIDS-defining disease [42]. According to sharing of immune-compromising mechanisms of both infections with* Leishmania infantum* and HIV-1 that may affect the parasite control in visceral leishmaniasis coinfected patients [42]. In comparison to patients with visceral leishmaniasis alone, coinfected patients present a more severe disease with increased parasite burden, frequent relapses, and antileishmanial drug resistance [41, 43]. On the other hand, Leishmania infection can impair both the chronic immune activation and the lymphocyte depletion and can accelerate progression to AIDS, particularly in HIV-1-infected individuals [44, 45]. Hence, serological testings for latent infection due to* Leishmania* species are indicated in the pretransplantation screening from endemic areas [46]. Immune activation can profoundly impact the visceral leishmaniasis clinical course and prognosis, leading to increase in the risk of death even under treatment of leishmaniasis [47]. Pentavalent antimonials; pentamidine; and amphotericin B, particularly the liposome formulations; and miltefosine are the drugs for the treatment of leishmaniasis [48]. A previous study demonstrated that Poly/hsp/pcDNA vaccine can significantly decrease parasite load in spleen and liver indicating a feasible, effective, and practical approach for visceral leishmaniasis [49].

## 5. Pulmonary Trypanosomiasis

Human African trypanosomiasis (HAT) or sleeping sickness is caused by an extracellularly protozoan parasite, called “*Trypanosoma brucei gambiense*” [50], “*Trypanosoma brucei rhodesie*” [51], and “*Trypanosoma cruzi*” which was discovered by Carlos Chagas in 1909 [52] and is endemic to West Africa and Central Africa, mostly in Democratic Republic of Congo, Angola, Chad, Central African Republic, Uganda, and Sudan [50]. HAT continues to threat more than 60 million people in 36 sub-Saharan countries [50, 53]. In mice model, hypercellularity and edema of alveolar walls, approximately 10 times thicker than normal alveolar wall, are identified and result in wall thickening although parasites are not demonstrated in alveoli [52]. Thickening and edema of bronchial walls of small and medium size bronchi due to parasite infiltration and significant inflammatory reaction (except large bronchi) in mice model were observed [52]. These bronchial inflammatory changes result in bronchial lumen reduction [52]. Most infected mice demonstrated infiltration of the walls of large blood vessels with extensive clusters of parasites in the myocytes of the muscular stratum and accompanied by an inflammatory reaction, interstitial edema, and rupture of muscle fibers [52]. These pathologically lung changes can contribute to pulmonary alveolar hemorrhage, bronchiolitis, and pneumonitis [52]. Pulmonary emphysema was also observed in the lungs of infected rats [54]. By the statistical analysis, the difference between experimental groups in lung-parasitic distribution and the degree of inflammatory reaction demonstrated no statistical difference [52]. Most Mexican strains demonstrated cardiomyotropism [52] and could cause pulmonary hypertension that could result in the dilatation of the right ventricle which is a typical characteristic of Chagas' disease caused by* Trypanosoma cruzi* without affecting the left ventricle [52]. However, many cases of Chagas' disease with pulmonary hypertension associated with right ventricular dilatation could attribute to left ventricular failure [55]. A previous study on treatment of relapsing trypanosomiasis in Gambian population demonstrated that a 7-day course of intravenous eflornithine was satisfactory and would result in substantial savings compared with the standard 14-day regimen although the prior regimen was inferior to the standard regimen and could be used by the national control programmes in endemic areas, provided that its efficacy was closely monitored, whereas melarsoprol remains the only effective therapeutic option for new cases [56]. In animal experimental studies, eflornithine and melarsoprol synergistically act against trypanosomes since the former drug decreases the trypanothione production, the target of the latter drug [8, 57]. A recent study demonstrated that oxidative stress could contribute to parasite persistence in host tissue and the development of anti-*Trypanosoma cruzi* drugs [58].

## 6. Pulmonary Larval Migrans

*Toxocara* larval migrans caused by* Toxocara canis*, a parasite in dogs' intestine, and* Toxocara cati*, a parasite in cats' intestine, infected intermediate host, humans, by ingestion of these embryonated* Toxocara* eggs which hatch into infective larvae in the human intestine [1]. The infective larvae then penetrate into the intestinal wall and are carried by blood circulation to many organs including lungs, liver, central nervous system, eyes, and muscles [1]. Granulomata then occur in these organs and later develop fibrosis and calcification [1]. Pulmonary manifestations are found in 80% and patients present with severe asthma [1]. Clinical manifestations may demonstrate scattered rales and rhonchi on auscultation including fever, cough, hepatosplenomegaly, generalized lymph node enlargement, eye pain, strabismus, white pupil, unilaterally visual loss, abdominal pain, and neurological manifestations [1]. Some cases may present with severe eosinophilic pneumonia and may contribute to respiratory distress syndrome [59–61]. Chest roentgenogram may demonstrate localized patchy infiltrates [1]. This syndrome is usually associated with eosinophilia, elevated antibody titers to* Toxocara Canis*, and increased total serum IgE level [62, 63]. About 25% of childhood patients have no eosinophilia [64]. Identification of serum IgE antibodies by ELISA [65] and* Toxocara* excretory-secretory antigens by Western-blotting method has been reported for diagnosis [66]. Nevertheless, serodiagnostic methods cannot distinguish between past and current infections [65, 66].* Toxocara* eggs or larvae cannot be identified in the feces since human is not the definitive host [1]. Histopathological examination of lung or liver biopsy specimens may reveal granulomas with multinucleated giant cells, eosinophils, and fibrosis [1].* Toxocara* larval migrans may be spontaneous resolution; therefore, mild to moderate symptomatic patients need not any treatment [1]. However, patients with severe* Toxocara* larval migrans can be treated with diethylcarbamazine (6 mg/kg/day, 21 days) [67], mebendazole (20–25 mg/kg/day, 21 days) [68], or albendazole (10 mg/kg/day, 5 days) [69]. Exacerbation of the inflammatory reactions in the tissues due to killing of the larvae may occur; therefore, antihelminthics plus corticosteroids is recommended [1].

## 7. Pulmonary Toxoplasmosis

A celled protozoan parasite, called “*Toxoplasma gondii*” which are primarily carried by cats, is causal microorganism [70]. Humans are infected by ingestion of parasitic cyst-contaminated uncooked milk product, vegetables, or meat [1]. The clinical manifestations are influenza-liked illness, myalgia, or enlarged lymph nodes [1] which is the most common recognized clinical manifestation [71]. Pulmonary involvement has been increasingly reported in HIV-infected/AIDS patients [1]. Pulmonary manifestations may be interstitial pneumonia, diffuse alveolar damage, or necrotizing pneumonia [72]. Nevertheless, obstructive or lobar pneumonia has been reported in a 49-year-old Spanish heterosexual man [71]. Early pregnancy with toxoplasmosis can cause fetal death and chorioretinitis and neurological symptoms in the newborn, whereas chronic disease can cause chorioretinitis, jaundice, convulsion, and encephalitis [1]. Diagnosis of toxoplasmosis is based on detection of the protozoan parasites in the body tissues [1]. Sputum examination was used in diagnosis of pulmonary or disseminated toxoplasmosis in a 14-year-old allogeneic bone marrow recipient with graft-versus-host disease by identification of* Toxoplasma gondii* tachyzoites in sputum smears [73]. Serodiagnosis is unable to discriminate between active and chronic* Toxoplasma gondii* infection due to ability to increase the antibody levels without active disease [1]. A real-time-PCR-based assay in BAL fluid has been performed in HIV-infected/AIDS patients [74]. Toxoplasmosis can be treated with a combination regimen of pyrimethamine and sulfadiazine [1].

## 8. Pulmonary Babesiosis

This disease is caused by haemoprotozoan parasites,* Babesia divergens* and* Babesia microti* [75]. Humans are infected by the bite of an infected tick,* Ixodes scapularis*, or by contaminated blood transfusion [76]. The parasites can attack the red blood cells and can contribute to misdiagnosis of* Plasmodium* [76]. The symptoms include fever, headache, loss of appetite, myalgia, tiredness, and drenching sweats [77]. Patients with babesiosis are frequently complicated by noncardiogenic diffuse-bilateral-interstitial pulmonary edema and adult respiratory distress syndrome [78]. Giemsa-stained thin blood smear examination, specific antibody detection, and PCR method are specific diagnosis of pulmonary babesiosis [75]. Treatment of choice is combination of clindamycin (600 mg every 6 hours) and quinine (650 mg every 8 hours) or atovaquone (750 mg every 12 hours) and azithromycin (500–600 mg on the first day and 250–600 mg on subsequent days) for 7–10 days [79, 80].

## 9. Filarial Parasites Associated with Tropical Pulmonary Eosinophilia

This syndrome results from immunological hyperresponsiveness to human filarial parasites,* Wuchereria bancrofti* and* Brugia malayi* [81]. Tropical pulmonary eosinophilia (TPE) is one of the main causes of pulmonary eosinophilia in the tropical countries and is prevalent in filarial endemic regions of the world particularly Southeast Asia [81, 82]. Clinical findings are cough, fever, chest pain, and body weight loss in association with massive blood eosinophilia [83]. Chest roentgenographs demonstrate military infiltrates of both lungs mimic miliary TB or not miliary infiltrates [84]. Additionally, there may be prominent hila with heavy vascular markings [85–88], but 20% of cases present with normal chest roentgenographs [89]. Some previous studies of computed tomographic scan of the chest demonstrated air trapping, mediastinal lymphadenopathy, calcification, and bronchiectasis [90]. At least 120 million people are globally infected with mosquito-borne lymphatic filariasis [89], but only less than 1% of filarial infection causes TPE [91], whereas various studies have demonstrated that filarial infection is the cause of TPE [81, 92]. A positive immediate reaction to intradermal skin tests with* Dirofilaria immitis* antigens has been demonstrated in patients with TPE [93]. Microfilariae, anatomical features of* Wuchereria bancrofti*, had been demonstrated in the lungs, liver, and lymph nodes of the patients with TPE [94–96] but are rarely identified in the blood [94]. Filarial specific IgG and IgE concentration elevation have been observed in TPE [97]. Peripheral basophils from patients with TPE released greater amounts of histamine when they were challenged with* Wuchereria* or* Brugia* antigens than with* Dirofilaria* antigen [97]. This indicated that TPE resulted from immunological hyperresponsiveness to human filarial parasites [97]. Leukocyte adhesion phenomenon in sera from patients with TPE using* Wuchereria bancrofti* revealed maximal positive results compared with* Dirofilaria immitis* and* Dirofilaria repens* [98]. Demonstration of living adult* Wuchereria bancrofti* in the lymphatic vessels of the spermatic cord of the patients with TPE is evidenced by ultrasound examination [99] and biopsy of a lump in the spermatic cord shows degenerating adult female filarial worm with uteri full of microfilariae [100]. There is a marked reduction of filarial-specific IgG and IgE levels in the lung epithelial lining fluid [101] and roentgenological improvement [102, 103] after 6–14 days of therapy with diethylcarbamazine citrate (DEC). The standard treatment recommended by the World Health Organization is oral DEC (6 mg/kg/day) for three weeks [104]. The usefulness of DEC in the treatment of TPE further focuses attention on its filarial etiology [105, 106].

## 10. Pulmonary Dirofilariasis

Human pulmonary dirofilariasis, a disease of middle-age adults [107], is caused mainly by immature filarial nematodes of* Dirofilaria immitis* [108–112] Other species,* Dirofilaria repens* and* Dirofilaria tenuis*, are known to infect humans [113]. These organisms, namely, dog heartworm [1], are usually transmitted from domesticated dogs or other carnivores (fox, wolf, cat, otter, muskrat, coyote, jackal, and sea lion) [113–115] to humans (accidental host) [114] by infected mosquitoes (*Aedes*,* Culex*, or* Anopheles*) [116], with a distribution in Asia, Australia, Southern Europe, and North and South Americas [114]. Majority of cases are frequently found in the lung periphery, particularly, the right lower lobe [113, 117, 118]. The lung lesion could be a solitary-coin nodule or multiple nodules, usually less than 3 cm in size [109], as noted on the chest roentgenogram [1, 114]. Pathologically, the lung lesion is demonstrated as a spherical infarct centered on the obstructive artery [113]. Clinical manifestations may include fever, chill, chest pain, hemoptysis, and malaise [1, 119–121]. At least 50% of the patients are asymptomatic [1, 113, 122]. Only 17% of the studied patients in the Japanese series were identified to have eosinophilia [122]. The serological tests, sputum cytological analysis, bronchial washing, and transthoracic needle biopsy provide low specificity for accurate diagnosis [123]. Definitive histopathological diagnosis could be achieved by wedge biopsy of tissue specimens through the video-assisted thoracoscopy [122] and polymerase chain reaction (PCR) methods [124, 125]. Wedge resection of the pulmonary nodule is usually curative without specific medical therapy [126]. Some suggestions have indicated the use of ivermectin with or without DEC for treatment of pulmonary dirofilariasis, but these suggestions are not widely accepted [127].

## 11. Pulmonary Strongyloidiasis

Very few parasitic diseases have been reported to cause pneumonia in HIV-infected/AIDS patients [128]. A helminth,* Strongyloides stercoralis* which is commonly found in many tropical and subtropical areas, has occasionally been reported as the cause of pulmonary disease [128]. The prevalence of this helminth in stool specimen varies from region to region as follows: 26–48% in Sub-Saharan Africa, 15–82% in Brazil, 1–16% in Ecuador, and 4–40% in the USA [129]. Although the prevalence of strongyloides infection in Southeast Asia is high, no cases have been reported in the English language literature [128]. There have been relatively few cases reported of helminth infection in AIDS patients in the tropics despite its high prevalence [128]. In a previous study in Brazil, 10% of 100 AIDS patients were infected with* Strongyloides stercoralis* [130], whereas in a study in Zambia, 6% of 63 HIV-infected patients with chronic diarrhea were infected with* Strongyloides stercoralis* [131]. The parasitic females live in the mucous membrane (wall) of small intestine of humans, particularly in the lamina propria of the duodenum and proximal jejunum, whereas the parasitic males remain in the lumen of the bowel and they have no capability to penetrate the mucous membrane [1]. Eggs are laid by female parasites and contain larvae ready to hatch [1]. The rhabditiform larvae emanating from the eggs pierce the mucous membrane and reach the lumen of the bowel [1]. These larvae are then passed with feces and can penetrate the intestinal epithelium or perianal skin without leaving the host by metamorphosing into filariform larvae in the lumen of small intestine [1]. This contributes to autoinfection and persistence of infection for 20 to 30 years in individuals who have left the endemic regions [132]. The rhabditiform larvae that are voided with the feces can undergo two distinct cycles in the soil: (1) direct (host-soil-host) cycle and (2) indirect cycle [1]. In direct cycle, the rhabditiform larvae directly metamorphose into filariform larvae and can infect humans through skin [1]. In indirect cycle, the rhabditiform larvae mature into free-living sexual forms (males and females) and then produce second generation of rhabditiform larvae [1]. These rhabditiform larvae are then transformed into filariform larvae and can directly penetrate through the skin, invade the tissue, penetrate into the lymphatic or venous channels, and are carried by the blood stream to the heart and lungs [1]. These filariform larvae pierce the pulmonary capillaries; enter the alveoli; migrate to the bronchi, trachea, larynx, and epiglottis; and then are swallowed back into the intestine [1]. In the duodenum and jejunum, these filariform larvae develop into sexual forms to continue the life cycle [1]. Following primary infection, the cell mediated immunity prevents reinfection by confining of the larvae and the adult form to the intestine and preventing the tissue invasion in immunocompetent persons, but autoinfection is exaggerated in immunosuppressed ones and contributes to hyperinfection [1]. During migration of filariform larvae through the lungs, bronchopneumonia and alveolar hemorrhages can occur [1]. The pathological lungs are infiltrated with eosinophils and are associated with elevated serum IgE and eosinophilia [1]. These pathological lung findings can be aggravated by the secondary bacterial infections [1]. Most patients with hyperinfection present with cough, fever, shortness of breath, and usually diffuse pulmonary infiltrates [133]. The definite diagnosis is to identify the helminth in the respiratory specimens or stool [133]. Previous reports demonstrated that at least two cases with strongyloides hyperinfection had concomitant pneumocystis [134, 135]. A previous review of the literature revealed that only surviving patients were treated with thiabendazole, 25 mg/kg twice a day for five days with three courses 10 days apart followed by monthly course of thiabendazole, whereas the duration of treatment in HIV-1 infected individual is unknown [135]. Generally, most patients have died directly or indirectly from their strongyloides hyperinfection [135]. It seems cautious to treat any patient who is infected with* Strongyloides stercoralis* detected in the stool despite the rarity of clinically significant strongyloides infection in HIV-infected/AIDS patients [135].

## 12. Pulmonary Ascariasis

*Ascaris lumbricoides* is the most common intestinal helminthic infection [136]. Both fertilized and unfertilized eggs are passed in the feces and released in the soil [137]. Infection occurs through soil contamination of hand or food with eggs and then swallowed [137]. The eggs hatch into larvae in the small bowel, which is called “first stage;” then, they moult into second-stage larvae in the lumen of the small bowel. The second-stage larvae penetrate the wall of the intestine and migrate via lymphatics and capillaries to the hepatic circulation and to the right side of the heart and then reach the lungs [137]. The second-stage larvae moult twice more in the alveoli to produce third- and fourth-stage larvae. The fourth-stage larvae which are formed 14 days after ingestion migrate upward to the trachea and then are swallowed to reach back the small bowel [137]. The fourth-stage larvae take approximately 10 days for migration from the lungs to the small intestine [137]. It takes 10–25 days to produce eggs after initial ingestion [137]. The migrating larvae can induce tissue-and lung-granuloma formation with macrophages, neutrophils, and eosinophils [138]. This may produce hypersensitivity in the lungs and result in peribronchial inflammation, increased bronchial mucus production, and finally, bronchospasm [138].* Ascaris lumbricoides* can produce both specific and polyclonal IgE [138]. Elevation of IgG4 levels in patients with Ascariasis has also been reported [139]. Symptomatic pulmonary involvement may range from mild cough to a Loffler's syndrome which is a self-limiting lung inflammation and is associated with blood and pulmonary eosinophilia, particularly childhood Ascariasis [3, 140]. This syndrome can occur as a result of exposure to various drugs. Clinical presentation may vary from malaise, fever, loss of appetite, myalgia, and headache [3, 140] to respiratory symptoms which include sputum-productive cough, chest pain, hemoptysis, shortness of breath, and wheezing [141]. Chest roentgenographic findings usually demonstrate peripherally basal opacities, but they occasionally show unilateral, bilateral, transient, migratory, nonsegmental opacities of various sizes [142]. Mebendazole (100 mg twice a day for three days or a single dose of 500 mg) and albendazole (single dose of 400 mg) have been observed to be equally effective in the treatment of ascariasis [1]. Pyrantel pamoate (a single dose of 11 mg/kg, maximum dose one gram) and piperazine citrate (50–75 mg/kg/day for two days) are also useful as well as ivermectin [1].

## 13. Pulmonary Ancylostomiasis

### 13.1. *Ancylostoma duodenale*

*Ancylostoma duodenale* can live only one year [143, 144]. Female* Ancylostoma duodenale* produces 10,000 to 30,000 eggs per day [143, 144]. Man is the only definite host [143, 144].* Ancylostoma duodenale *larvae can enter the human host via the oral route in addition to the skin and it can reach pulmonary circulation through the lymphatics and venules [143].* Ancylostoma duodenale *larvae can developmentally get arrested in the intestine or muscle and restart development when environmental conditions become favorable [145]. Bronchitis and bronchopneumonia can occur when the larvae break through the pulmonary capillaries to enter the alveolar spaces [128, 143, 144]. Pulmonary larval migration can develop peripheral blood eosinophilia [128, 143, 144]. Hookworm larvae can release a family of protein called “ancylostoma-secreted proteins (ASP)” [128, 143, 144] and can secrete low-molecular weight polypeptides which inhibit clotting factor Xa and tissue factor VIIa [146]. During pulmonary larval migration, the patients may present with cough, fever, wheezing, and transient pulmonary infiltrates that are associated with blood and pulmonary eosinophilia [128]. Both albendazole (single dose of 400 mg) and mebendazole (100 mg twice daily for three days) are drugs of choice for treatment of hookworm [128]. Pyrantel pamoate (single dose of 11 mg/kg with maximum dose of 1 g, orally) is an alternative drug of choice [128]. A previous study revealed that ivermectin can effectively treat hookworm infections [128].

### 13.2. *Necator americanus*

*Necator americanus* larvae can infect human only through the skin [143]. The larvae reach the lungs with the same mechanisms as the* Ancylostoma duodenale* [128]. The interval between the time of skin penetration and laying of eggs by adult worms is about six weeks [128]. Bronchitis and bronchopneumonia can occur when the larvae break through the pulmonary capillaries to enter the alveoli [128]. Drugs of choice for treatment of* Necator americanus* are the same as the drugs of choice for treatment of* Ancylostoma duodenale* [128].

## 14. Pulmonary Paragonimiasis

In Asia, nearly 20 million people are infected with* Paragonimus* species such as* Paragonimus westermani* which is the main species in humans,* Paragonimus mexicanus*,* Paragonimus africanus*,* Paragonimus miyazakii*,* Paragonimus philippinensis*,* Paragonimus kellicotti*,* Paragonimus skrjabini*,* Paragonimus heterotremus*, and* Paragonimus uterobilateralis* [147–149]. Paragonimiasis is a foodborne zoonoses [128]. Humans get* Paragonimus* species when they ingest raw crayfishes or crabs infected with infective metacercariae [147]. The incubation period may vary from 1 to 2 months or even longer [150]. The shorter incubation period from 2 to 15 days can occur [150]. The parasite from the human gut passes through several organs and tissues to reach the lungs [147]. Adult worms live in the lungs and the eggs are voided in the sputum or feces [147]. The eggs hatch in the fresh water to release miracidiae which are ingested by the first intermediate host, fresh water snails [147]. The miracidiae develop into cercariae in the snail and are released into the water [147]. The cercariae then invade the second intermediate host, crustaceans (crayfish or crabs), and develop into infective metacercariae [147]. Humans get infection, when they eat undercooked or raw crabs or crayfishes infected with infective metacercariae [151]. The infective metacercariae from the human intestine passes through several organs and tissues to reach the lungs [147]. The migrating worms in the pleural cavity produce effusion and pleuritis [150]. Pulmonary paragonimiasis is the commonest form of paragonimiasis [150] that is initially manifested as coughing up rusty brown or blood-stained sputum or recurrent hemoptysis, chest pain, fever, chest tightness, difficulty in breathing, mild pleural effusion, bronchiectasis, pneumonitis, or bronchopneumonia [150, 152]. Generally, most patients are ambulatory and apparently healthy [150]. The chronic pulmonary form may be associated with fever, weakness, weight loss, and anemia [150]. Generally, pulmonary paragonimiasis has high morbidity and low mortality unless being complicated with infection in the vital organs [150]. Cough, hemoptysis, fever, and chest pain were observed in the* Paragonimus* ova-positive patients, particularly cough and hemoptysis [153]. In children with tuberculosis, difficulty in breathing is noted, but not in those with paragonimiasis [153]. This symptom is usually present in severe case associated with pleural effusion or coinfected with tuberculosis [153]. There is no difference noted in most of the chest signs of both paragonimiasis and tuberculosis [153]. Presence of crepitations in children with tuberculosis is a significant finding; however, they may be detected in acute phase of pulmonary paragonimiasis [153]. Both adult and children with pulmonary paragonimiasis have similar symptoms, including lethargy [153]. A recent study demonstrated that all children with pulmonary tuberculosis had roentgenographic evidence of subcutaneous tissue wasting [153]. Therefore, this roentgenographic sign is a critical differentiating factor in addition to bacteriological investigations in an area where both paragonimiasis and tuberculosis exist [153]. Pneumothorax or pleural effusion (usually bilateral) is an important manifestation in paragonimiasis [150, 154]. Chest roentgenographs may demonstrate infiltrates, patchy air-space consolidation, or opacity associated with pleural reaction or thickening, single or multiple nodules, cystic lesions, cavities, pleural thickening, and nodular opacities [150, 155]. The chest roentgenograms demonstrate patchy consolidation in 62–71%, pleural thickening in 28%, pleural effusion in 9-10%, nodular lesions in 8–13%, and cystic or cavitary lesions in 11–14% [150]. Nevertheless, normal chest roentgenogram may occur in symptomatic pulmonary paragonimiasis [150] and may be identified approximately in 8%–20% of patients with late-stage paragonimiasis [156–158]. Persistent bilateral pleural effusion was demonstrated in 44.4% in a study conducted in Lao PDR [150]. Oloyede et al. reported that the roentgenographic features of both pulmonary paragonimiasis and pulmonary tuberculosis identified in their study were mostly parenchymal and were similar [153]. The main differential diagnosis of cavitating pulmonary infiltrates includes bacterial abscess, tuberculosis, fungal infections, nocardiosis, and parasitic lung diseases [157]. Computerized tomography of the chest is found as a better technique compared to the chest roentgenogram by visualization of burrows and tunnels joining the* Paragonimus* cystic lesions [150]. The parasitic eggs can be shown in sputum specimens, bronchoalveolar lavage fluid, or lung biopsy specimens [159]. Endobronchial lesions can occur in approximately 54% of patients with pulmonary paragonimiasis [156]. Extrapulmonary paragonimiasis is likely to occur more commonly in heavy infected cases and children [150]. In adults, extrapulmonary form is found in only 2% [150]. Previous studies demonstrated* Paragonimus* ova-positive sputum in 55.6% to 72% of sputum specimens of patients with pulmonary paragonimiasis [150]. A study in adults and children with pleuropulmonary paragonimiasis demonstrated ova-positive sputum in 20.9% and 4.1%, respectively [150]. Nevertheless, this finding is unusual because sensitivity of sputum examination is expected to be higher in adults than in children [150].* Paragonimus* ova may be demonstrated in the centrifuged deposit of pleural fluid in approximately 10% of cases presented with pleuropulmonary paragonimiasis [150]. Pleural fluid analysis usually demonstrates low pH, eosinophilia, high-protein levels, lactose dehydrogenase higher than 1,000 IU/L, and glucose content less than 10 mg/dL [150]. Examination of two to three stool samples collected at consecutive days is recommended in children who usually swallow sputum and in patients whose sputum samples are apparently negative for ova [150].* Paragonimus* ova are identified in the fecal samples of the patients with pulmonary paragonimiasis in approximately 65.1% by AMS III concentration technique [150]. Peripheral blood eosinophilia and elevated serum IgE levels are demonstrated in more than 80% of cases with paragonimiasis [147, 154].* Paragonimus westermani* adult excretory-secretory products are composed of cysteine proteases which are involved in immunological reactions during parasitic infection [160, 161]. Many immunodiagnostic techniques have been introduced, such as intradermal test, immunodiffusion, complement fixation test, enzyme-linked immunosorbent assay (ELISA), dot-ELISA, and Western blot [150]. While a negative intradermal test almost certainly excludes paragonimiasis, a positive test cannot differentiate between the past and the present infection as the test may remain positive as long as 10 to 20 years even after the successful treatment or spontaneous recovery [150]. This test also reacts with other trematode infections, such as schistosomiasis and clonorchiasis [150]. The ELISA techniques are currently most widely used for serological diagnosis of paragonimiasis due to their high sensitivity and specificity [150]. Nevertheless, ELISA techniques are time-consuming and more expensive and they require costly equipment and experienced personnel and all antigens and reagents are not commercially available [150]. A rapid test which was developed in China has the sensitivity and specificity up to 99% and 92%, respectively [150]. Immunoglobulin G4 antibodies to an excretory-secretory product of* Paragonimus heterotremus* had accuracy, sensitivity, specificity, and positive and negative predictive values of 97.6%, 100%, 96.9%, 90%, and 100%, respectively [162]. In case of pleural or pleuropulmonary paragonimiasis, pleural fluid eosinophilia or peripheral blood eosinophilia is reported with incidence, but a small proportion of patients do not have these characteristic findings [163]. The measurement of pleural fluid adenosine deaminase levels can facilitate the diagnosis of tuberculous pleural effusion [163]. Excision biopsy may be served as surgical treatment of subcutaneous nodules as well as laboratory diagnosis [150]. Pathological examination will demonstrate* Paragonimus* ova, adult or immature worm, eosinophils, inflammatory cells, and Charcot-Leyden crystals [150]. Paragonimiasis can be treated with high-dose praziquantel (75 mg/kg/day for three days, approximately 2% relapse rate, approximately 100% cure rate for five-day treatment [150]), triclabendazole (20 mg/kg in two equal doses), niclofolan (2 mg/kg as a single dose), or bithionol (30 to 40 mg/kg in 10 days on alternative days) [147, 164, 165]. Control trials have demonstrated that triclabendazole, 10 mg/kg, single dose, had comparable safety, efficacy, and tolerability with praziquantel [150].

## 15. Pulmonary Schistosomiasis

*Schistosoma* species that cause human disease are* Schistosoma hematobium*,* Schistosoma japonicum*, and* Schistosoma mansoni* [166]. The schistosome eggs are passed in feces (*Schistosoma japonicum* and* Schistosoma mansoni*) or in urine (*Schistosoma hematobium*) [128]. The infective cercariae in water are ingested to penetrate the human gut or penetrate human skin and finally reside at the mesenteric beds (*Schistosoma japonicum* and* Schistosoma mansoni*) and the urinary bladder vesicle beds (*Schistosoma hematobium*) [128]. The life cycle of* Schistosoma* involves two hosts: humans and snails [166]. The eggs released by infected human in fresh water via feces or urine are then ingested by intermediate host, snail, in which the eggs hatch and go through several cycles and develop into cercariae in the water [128, 166]. The infective cercariae penetrate human skin or are ingested to penetrate the intestine [128, 166]. Persons mostly become infected while swimming in the contaminated water [166]. The schistosomule, the form of cercariae without tails, migrate first to the lungs and reach the liver within one week [166]. Approximately 6 weeks after reaching the liver, they mature into adult flukes that descend via the venules to their final habits: the mesenteric beds in the case of* Schistosoma mansoni* and* Schistosoma japonicum* and the urinary vesicle beds in the case of* Schistosoma hematobium* [166]. The life span of the adult fluke is known to be up to 30 years [166]. Most of the eggs laid by an adult fluke that are excreted by the host via feces or urine are significantly public health standpoint due to their ability of spreading of the disease [166]. A few of these eggs remain in the host tissue and can cause granuloma formation around them which are the causes of the clinical signs and symptoms of schistosomiasis [166]. Pulmonary schistosomiasis can clinically be present as acute or chronic form [128]. Acute manifestations, called “Katayama syndrome” or “Toxemic Schistosomiasis,” can develop three to eight weeks after skin penetration [159, 166–168].

The acute form is presented with dry cough, wheezing, shortness of breath, chill, fever, headache, malaise, weight loss, abdominal pain, diarrhea, urticarial, marked eosinophilia, arthralgia, myalgia [166, 167, 169], and several small pulmonary nodules ranging from 2–15 mm in size and larger nodules with ground glass-opacity halo [170] and ill-defined borders in the chest roentgenographs or computed tomography in immunocompromised patients, or diffuse, increased pulmonary markings and prominent hilum with ill-defined nodules which are less common patterns [166, 171]. The incidence of pulmonary involvement in the early stages of Schistosomiasis is unknown, but coughing was reported in more than 40% of the infected travelers [166]. This acute period is the stage at which the schistosomule mature into the adult form [166]. It has been reported that pulmonary involvement may occur in the early stage, particularly in nonimmune patients [166]. The physical examination is usually unremarkable, although demonstrating prolonged expiration in some patients [166]. The laboratory findings usually reveal 30%–50% of serum eosinophils with mild leukocytosis [166]. Occasional abnormal liver function test results and elevation of serum IgE are also noted [166]. Patients with chronic form present with pulmonary hypertension and cor pulmonale [172, 173], whereas massive hemoptysis and lobar consolidation and collapse have been reported [174]. The sensitivity of the traditional tests of stool and urine examinations for parasite eggs are low, even when performed on three separate occasions [166]. The sporadic passage of parasite eggs by the adult flukes and the low fluke burden limit these tests' sensitivity, but rectal biopsy may increase the sensitivity [166]. Nonimmune patients may be acutely ill with pulmonary involvement before oviposition by the adult flukes has begun [166]. The serologic testing is much more sensitive [166]. By confirmation and speciation of positive enzyme-linked immunosorbent assay results performed with an enzyme-linked immunoelectrotransfer blot, the specificity in confirmation of the presence of the* Schistosoma* species can reach almost 100% [166]. This test usually yields positive results 4–6 weeks after parasite exposure [166]. However, the major limitation of the serologic test is that it stays positive for many years despite successful treatment, which excludes its use in confirming reexposure or assessment of the treatment outcome [166]. A good travel history of the patients, particularly regarding the exposure to freshwater in endemic areas, is the most critical clue to obtain the diagnosis [166]. Hepatosplenomegaly due to portal hypertension has been reported in patients infected with* Schistosoma japonicum* and* Schistosoma mansoni* [166]. In cases with chronic pulmonary involvement, the pathophysiologic mechanisms can be explained as follows. In case of* Schistosoma hematobium* infection, the final habitat of the adult fluke is in the perivesical plexus, in which the eggs were laid by the mature flukes [166]. The ectopic migration of the eggs can occur by egg sweeping through the systemic venous system to reach the lungs [166]. In case infected with* Schistosoma mansoni* and* Schistosoma japonicum*, the eggs are swept with the portal blood flow and are lodged in the venules of the liver, creating a granuloma around the eggs, which finally causes periportal hepatic fibrosis and portal hypertension [166]. Portal hypertension results in opening the portocaval shunts, which enables the eggs to be swept and lodged in the lungs [166]. Demonstration of* Schistosoma mansoni* eggs in the lungs without evidence of hepatic fibrosis has been documented [166]. The eggs trigger a granulomatous response that affects the intima and later the media wall of the pulmonary arteries, which results in obliterative arteritis, fibrosis, pulmonary hypertension, and later, the development of cor pulmonale [166, 170]. Lung histologic findings demonstrate dumbbell-shaped interarterial and perivascular granulomas with local angiogenesis, which causes dilated and twisted vessels, named “angiomatoid” [166]. The eggs either remain in the lumen of the pulmonary beds or migrate to the lung tissue itself [166]. The clinical features of chronic pulmonary schistosomiasis can be classified to three groups: (1) asymptomatic cases with schistosomal eggs in the pulmonary beds, with or without granuloma formation, (2) granuloma with pulmonary hypertension, and (3) granuloma formation with pulmonary hypertension and cor pulmonale [166]. It is evident that* Schistosoma hematobium* or* Schistosoma japonicum* causes much less cor pulmonale compared to* Schistosoma mansoni* [175] although* Schistosoma hematobium* is identified in pulmonary beds at significant percentages [166]. In chronic form, peripheral blood eosinophilia with mild leukocytosis, IgE levels, and abnormal liver function test had been reported [166]. Diagnosis of chronic schistosomiasis is based on the identification of eggs in stool or urine by direct microscopy or rectal/bladder biopsy [1]. Chest roentgenogram shows enlargement of the right ventricle, dilatation of the pulmonary arteries and trunk as well as their interlobar branches that indicates pulmonary hypertension and cor pulmonale [176]. Bronchoscopic examination and tissue biopsy are unlikely to be useful because of the small amount of the tissue obtained and the sporadic distribution of the granulomas [166]. The open lung biopsy demonstrated the diagnosis in some case reports, but this should not be adopted as protocol [166]. PCR testing of sputum demonstrated promising results, while sputum examination for the parasite eggs has a low yield [166]. Both acute and chronic schistosomiasis can be treated with praziquantel (20–30 mg/kg orally in two doses within 12 hours) and then praziquantel is repeated several weeks later to eradicate the adult flukes [166]. A short course treatment of steroids is effective in acute form before the beginning of praziquantel treatment and can be treated with artemether, an artemisinin derivative which acts on the juvenile forms of the schistosome [166]. A patient with schistosomiasis-associated pulmonary hypertension was reported of successful treatment with a phosphodiesterase-5 inhibitor [176].

## 16. Pulmonary Hydatid Disease

Human hydatid disease is caused by* Echinococcus multilocularis*,* Echinococcus granulosus*,* Echinococcus vogeli*, and* Echinococcus oligarthrus* [128, 177].* Echinococcus multilocularis* causes alveolar or pulmonary echinococcosis (AE),* Echinococcus granulosus* causes cystic echinococcosis,* Echinococcus vogeli* causes polycystic echinococcosis, and* Echinococcus oligarthrus* is an extremely rare cause of human echinococcosis [177]. The adult* Echinococcus multilocularis* resides in the small bowel of the definitive hosts, foxes, dogs, cats, coyotes, and wolves [177]. Gravid proglottids release eggs that are passed in the feces [177]. After ingestion by a suitable intermediate host (small rodents), the egg hatches in the small bowel and releases an oncosphere that penetrates the intestinal wall and migrates through the circulatory system into various organs, particularly the liver and lungs [128, 177]. In these organs, the oncosphere develops into a cyst that enlarges gradually, producing protoscolices and daughter cysts that fill the cyst interior [177]. The larval growth in the liver indefinitely remains in the proliferative stage, resulting in invasion of the surrounding tissues [177]. The definite host is infected by ingesting the cyst-containing organs of the intermediate host [177]. After ingestion, the protoscolices evaginate, attach to the intestinal mucosa, and develop into adult stages within 32–80 days [177]. The same life cycle occurs with* Echinococcus granulosus*, with the following differences: the definitive hosts are dogs and other canidae and the intermediate hosts are sheep, goats, swine, and so forth [177]. With* Echinococcus vogeli*, the definitive hosts are bush dogs and dogs, the intermediate hosts are rodents, and the larval stage (in the liver, lungs and other organs) develops both externally and internally, resulting in multiple vesicles [177]. With* Echinococcus oligarthrus*, the life cycle involves wild felids as definitive hosts and rodents as intermediate hosts [177]. Humans become infected by ingesting embryonated eggs in feces of the definitive hosts [177]. Pulmonary alveolar echinococcosis is caused by hematogenous spreading from hepatic lesions [178]. The adult* Echinococcus granulosus* resides mainly in the small gut of the dogs [128]. The intermediate hosts including humans are infected by ingestion of parasitic eggs excreted in the feces of the dogs [128]. Clinical pulmonary manifestations include cough, dyspnea, chest pain, and fever [128]. Rupture of hydatid cysts into a bronchus may result in expectoration of cystic fluid containing parasite membrane, hemoptysis, asthma-liked symptoms, respiratory distress, persistent pneumonia, anaphylactic shock, and sepsis [179, 180] and elevation of serum IgG and eosinophilia [181]. Rupture of the echinococcal cysts into the pleural space may result in pleural effusion, empyema, and pneumothorax [1]. Immunodiagnostic tests using purified* Echinococcus granulosus* antigens have preferable sensitivity and specificity for the diagnosis of AE [182]. A serologic method using the synthetic p176 peptide for diagnosis of pulmonary hydatinosis demonstrated overall 78.69% of sensitivity and 96.88 of specificity [183]. The most significant factor associated with seropositivity is the presence of the hydatid cyst complications or rupture due to isolation of the hydatid cyst content from the human immune system by developing a very thick collagen layer, contributing to minimal or nil antigen release and subsequent minimal or nil antibody responses [183]. Chest roentgenographs demonstrate solitary or multiple round opacification mimicking lung tumors [184]. Air-fluid level, water-lily sign, Cumbo's sign (onion peel sign), and crescent sign are demonstrated in chest roentgenogram and computed tomography of the chest [1]. Signet-ring sign, serpent sign, and inverse crescent sign are demonstrated as the features of the pulmonary echinococcal cysts in computed tomography of the chest [1]. It has been experimentally revealed that magnetic resonance imaging can detect early pulmonary AE [185]. Unusual presentation of endobronchial hydatid cyst with a whitish-yellow gelatinous membrane was demonstrated by bronchoscopic examination in a child [186]. Treatment for many years with mebendazole, praziquantel, or albendazole is useful, particularly in inoperably recurrent and multiple cysts, but treatment of hydatid cyst is primary surgical [187]. The treatment of AE is radical surgical resection of entire parasitic lesion [187] but should avoid segmentectomy, lobectomy, and pneumonectomy [151, 188, 189].

## 17. Pulmonary Trichinellosis

The most important species that infects humans is* Trichinella spiralis* [190]. Humans get parasitic infection from ingestion of raw and infected pig's muscle or meat from wild animals such as bear containing larval* Trichinella* [191, 192]. The larvae develop into adults in the duodenum and jejunum [191]. Fertilized female worms release first-stage larvae into the bloodstream and the lymphatics [192]. Most of the newborn larvae penetrate into the submucosa and are carried in the circulatory and lymphatic systems to various organs, including lungs, myocardium, brain, lymph nodes, pancreas, retina, and cerebrospinal fluid [193]. The larvae undergo encystment in the muscle and a host capsule develops around the larvae and later on may get calcified [191]. Clinical pulmonary features include cough, dyspnea, and pulmonary patchy infiltrates on the chest roentgenographs [192, 194, 195]. The roentgenographic findings may include exaggerated and fuzzy lung markings and hilar enlargement [194]. Dyspnea is caused by parasitic invasion of the diaphragm and the accessory respiratory muscles [192]. The important laboratory findings are elevation of serum aminotransferase; serum aldolase; serum lactate dehydrogenase; and serum creatine phosphokinase, leukocytosis, and eosinophilia [195]. An enzyme-linked immunosorbent assay (ELISA) for identification of anti-*Trichinella* antibodies using excretory-secretory antigens [196] or anti-*Trichinella* IgG antibodies [192] may be useful in the diagnosis of* Trichinella spiralis* infection; a definite diagnosis can be performed by muscle biopsy (preferably deltoid muscle) [195]. Treatment of choice is with mebendazole, 200 to 400 mg, three times per day for three days followed by 400 to 500 mg, three times per day for 10 days [128]. The alternative drug of choice is albendazole, 400 mg per day for three days followed by 800 mg per day for 15 days [128]. Symptomatic treatment of trichinosis is analgesics and corticosteroids [128, 192].

## 18. New Emerging Human Parasites

A number of new human parasites have urged the interest of scientific community in addition to many zoonotic potential parasites [197]. Potential human parasites transmitted from wildlife domestic hosts are* Dirofilaria ursi*,* Dirofilaria subdermata*,* Dipetalonema* species,* Loa Loa*,* Thelazia callipaeda*,* Babesia duncani*,* Babesia venato rum*,* Babesia*-liked organisms,* Babesia divergens*-liked organism,* Leishmania braziliensis*,* Leishmania major*,* Plasmodium knowlesi*,* Plasmodium simium*,* Onchocerca gutturosa*,* Onchocerca cervicalis*,* Onchocerca jakutensis*,* Onchocerca dewittei japonica*,* Onchocerca lupi* [197, 198],* Diphyllobothrium dendriticum* [199], and so forth. Macaques (*Macaca fascicularis*) are believed to be the primary hosts of* Plasmodium knowlesi* [200], identified by PCR in58% of human malaria cases in the Kapit division of Sarawak, Malaysia, between 2000 and November 2002 [198]. It is transmitted by the forest dwelling* Anopheles latens*, a mosquito species that feeds on both themonkey natural host as well as on humans [197]. A single human malaria case of* Plasmodium knowlesi*, confirmed by PCR, has been reported from Thailand [198]. Outbreaks of Chagas disease transmitted by accidental ingestion of* Trypanosoma cruzi*-contaminated guava, sugar cane or acai' juices have been reported in South America [197].

## 19. Conclusions

Investigation of travel history is significant as this may indicate parasitic infection such as tropical eosinophilia,* Leishmania*, malaria,* Schistosoma*,* Trypanosoma*,* Toxocara*, or amoebiasis. Exposure to cats or dogs may indicate* Toxocara* or* Ancylostoma* infection. Multiple organ involvement may indicate Churg-Strauss syndrome. Clinical assessment must exclude other interstitial lung diseases and both primary and secondary lung malignancies. Education programs should be carried out in endemic areas for more awareness of the risk of oral transmission of* Trypanosoma cruzi*. Control measures are certainly not enough to stop the spreading of visceral leishmaniasis since global changes are accelerating this process. The real impact of global changes on the ecoepidemiology of the leishmaniasis in both traditional and nontraditional endemic areas might be unpredictable. Identification of priorities for society's scientific resources that are beneficial for global ecosystem integrity and human health and well-being at this complex intersection of humans, wildlife, domestic animals, and pathogens is difficult, but this problem can be solved by encouraging proactive exploration and assessment of potentially major influences on parasite flow from domestic animals and wildlife to humans, particularly environmental and climate changes, human intrusion into domestic and wildlife habitats, and other potential causes of ecosystem disruption. In arthropod or vector-borne parasite control, monitoring environmental and climatic changes remain a significant issue to be considered since these factors may affect both ecology and behavior of arthropod vectors. It is recommended that the national public health surveillance services should perform systematic entomological surveys in vector-free areas that are at risk of introduction of vectors through human or animal populations. Summary of parasitic diseases, chest roentgenographic features, and chemotherapeutic agents is demonstrated in Table 1.

## Figures and Tables

**Table 1:** Parasitic diseases, chest roentgenographic features, and chemotherapeutic agents.

Disease	Chest roentgenographic features	Reference	Chemotherapeutic agents	Reference
Malaria	Diffuse interstitial pulmonary edema, pleural effusion, lobar consolidation, bilaterally pulmonary infiltrates, diffuse bilateral alveolar opacities, bilateral basal ground glass opacities	[12–15]	Chloroquine (all *Plasmodium* species), Artemisinin-based combination regimens (all *Plasmodium* species)	[1, 11, 20, 22–25]

Amoebiasis	Pleural effusion, basal pulmonary involvement, elevation of hemidiaphragm	[1]	Metronidazole, diloxanide, lactoferrin, lactoferricin	[34, 35]

Leishmaniasis	Pleural effusion, mediastinal lymphadenopathy, pneumonitis (immunocompromised status)	[39, 40]	Pentavalent antimonials, pentamidine, amphotericin B, miltefosine	[48]

Trypanosomiasis	Pulmonary alveolar hemorrhage, alveolitis, pneumonitis, pulmonary emphysema (*in vivo*)	[52, 54]	Eflornithine, melasoprol (*in vivo*)	[8, 56, 57]

Pulmonary larval migrans	Localized patchy infiltrates	[1]	Diethylcarbamazine, Mebendazole, Albendazole	[67–69]

Toxoplasmosis	Interstitial pneumonia, diffuse alveolar damage, necrotizing pneumonia, obstructive or lobar pneumonia	[71, 72]	Combination regimen of pyrimethamine and sulfadiazine	[1]

Babesiosis	Noncardiogenic diffuse-bilateral-interstitial pulmonary edema and adult respiratory distress syndrome (complicated case)	[78]	Combination of clindamycin and quinine, or atovaquone and Azithromycin	[79, 80]

Filariasis	Bilateral military infiltrates, prominent hila with increased lung markings, normal	[84–89]	Diethylcarbamazine	[101–106]

Dirofilariasis	A solitary-coin or multiple nodules (usually less than 3 cm. in size, usually in the periphery of the right lower lobe)	[1, 109, 113, 114, 117, 118]	No specific medical therapy, but ivermectin may be useful, usually curative by wedge resection of the pulmonary nodule	[126, 127]

Strongyloidiasis	Bronchopneumonia, alveolar hemorrhages	[1]	Thiabendazole	[135]

Ascariasis	Peripherally basal opacities, unilateral or bilateral transient-migratory-non-segmental opacities of various sizes	[142]	Mebendazole, albendazole, pyrantel pamoate, piperazine citrate, ivermectin	[1]

Ancylostomiasis	Bronchitis, bronchopneumonia, transient pulmonary infiltrates	[128, 143, 144]	Mebedazole, albendazole, pyrantel pamoate, ivermectin	[128]

Paragonimiasis	Patchy consolidation, pleural thickening, pleural effusion, nodular lesions, cystic lesions, cavities, normal	[150, 153, 155–158]	Praziquantel, triclabendazole, niclofolan, bithionol	[147, 150, 164, 165]

Schistosomiasis	Multiple ill-defined small nodular lesions with ground glass-opacity halo, prominent hila, increased lung markings, enlargement of the right ventricle, dilatation of the pulmonary arteries and trunk as well as their interlobar branches (pulmonary hypertension and cor pulmonale)	[166, 170–173, 176]	Praziquantel, artemisinin derivatives	[166]

Hydatidosis/echinococcosis	Solitary or multiple round opacities with air-fluid level, water-lily sign, onion-peel sign, crescent sign	[1, 184]	Praziquantel, mebendazole, albendazole,	[187]

Trichinellosis	Patchy infiltrates, exaggerated and fuzzy lung markings, hilar enlargement	[192, 194, 195]	Mebendazole, albendazole	[128, 192]
